# Development and characterisation of orally disintegrating flurbiprofen tablets using SeDeM-ODT tool

**DOI:** 10.1371/journal.pone.0309894

**Published:** 2024-10-29

**Authors:** Syeda Sara Fatima, Farya Zafar, Huma Ali, Fahim Raees, Ghazala Raza Naqvi, Shazia Alam, Riffat Yasmin, Anum Tariq, Rehana Saeed, Sohail Khan

**Affiliations:** 1 Department of Pharmaceutics, Faculty of Pharmacy and Pharmaceutical Sciences, University of Karachi, Karachi, Pakistan; 2 Institute of Pharmaceutical Sciences, Jinnah Sindh Medical University, Karachi, Pakistan; 3 Department of Mathematics, NED University of Engineering and Technology, Karachi, Pakistan; 4 Department of Pharmaceutics, Faculty of Pharmacy, Federal Urdu University of Arts, Science &Technology, Karachi, Pakistan; 5 Department of Pharmaceutics, College of Pharmacy, Ziauddin University, Karachi, Pakistan; 6 Dow College of Pharmacy, Dow University of Health Sciences, Karachi, Pakistan; Bahauddin Zakariya University, PAKISTAN

## Abstract

In this study, SeDeM–ODT parametric tests were performed to determine the use of ludipress as a directly compressible tableting excipient for the development of a flurbiprofen orally disintegrating tablet. The preformulation features of different formulations (F1 –F9) were analyzed by the SeDeM–ODT tool which showed that all the powder blends were appropriate for direct compression since all the blends had index of good compressibility and bucodispersibility (IGCB) values above 5, signifying direct compression is the most appropriate method. The powder blend of the optimized formulation was assessed by the DSC–TGA technique. The optimization of nine different formulations blends of orally disintegrating tablets (ODTs) was prepared in various ratios by the implementation of design of experiments (DoE), using the central composite design by selecting ludipress (X_1_) (49–55%) and croscarmellose sodium (X_2_) (1–5%) while hardness, friability, and disintegration tests were selected as responses. The optimized formulations were evaluated by various tests and the results indicated that all the formulations were found to be in adequate range. Formulations were subjected to stability studies at accelerated states following ICH guidelines. Shelf life was found to be 51.144–56.186 months. Results of multiple-point dissolution studies revealed that formulations followed the Higuchi kinetic model. This study revealed that the SeDeM—ODT tool has been successfully used to determine the compression behavior of active compounds and their powder blends for the direct compression (DC) method in formulating flurbiprofen–ODT tablets.

## Introduction

Tablets are conventional dosage forms widely used for good patient compliance, however, some of the groups in the population i.e., pediatrics and geriatrics encounter difficulty in swallowing (dysphagia). In the 1990s, researchers introduced orally disintegrating tablets meant to be dispersed in the mouth [1]. According to European Pharmacopeia ODTs should disintegrate in less than 3 minutes [2]. ODTs are rapidly dissolving tablets that augment the effect of the drug in the pre-gastric region and therefore, increase the bioavailability of the drug by avoiding first-pass metabolism. ODTs provide rapid dispersion and are easily swallowed in liquid forms [3]. Various methods are used to manufacture ODTs but the direct compression technique has been extensively used due to the lesser number of production steps also it is suitable for hydrophobic and thermosensitive active pharmaceutical ingredients and enables a rapid dissolution process as the drug disintegrates rapidly. In the direct compression technique, excipients have to compensate for the poor flowability and compressibility associated with active pharmaceutical ingredients. For this reason, a new system i.e., the SeDeM ODT expert system was introduced which reduces the number of experiments and predicts the suitability of the substances used in the formulation [4].

Design of experiments (DoE) has been widely used to comprehend the effects of multidimensional interactions of input factors on the output variables of pharmaceutical products. It is a statistical tool that is used for determining the relation between the dependent and independent variables providing quick optimized outcomes [5].

The SeDeM-ODT expert system is a helpful tool in predicting the parameters of formulation for direct compression. This system uses a pre-formulation technique for evaluating the formulation by minimizing the number of experiments for industrial production. This system is applied to powder blends. It is mainly based upon the quantitative analysis and experimental methods for the characterization of the incidences of powder blends. For the SeDeM ODT expert system, 15 parameters are evaluated. This tool aims to provide information on raw materials for predicting whether it is appropriate for the direct compression method. The graphical representation of the pre-formulation studies gives an overview of different powder bends. By implementing different compendial and non-compendial methods for assessment that are commonly used in industries. SeDeM generates distinctive profiles for powder blends, excipients, and API. This profile for powder blends is used for predicting the suitability of the excipients and API to get adequate ratios for direct compression of tablets [6]. Various parameters are evaluated for the suitability of direct compression and bucco-dispersibility in the SeDem ODT expert system. Powder blends are compressed to ODTs with super disintegrant and subjected to compendial and non-compendial quality control tests [7].

In the present work, a directly compressible method using a SeDeM–ODT tool–driven central composite design was used to develop flurbiprofen orally disintegrating tablets (ODTs) using varying concentrations of ludipress and crospovidone. The developed formulations were characterized and evaluated by various compendial and non-compendial methods.

## Experimental

Flurbiprofen was obtained as a gift sample from Platinum Pharmaceuticals (Pvt.) Ltd. Ac-di-sol and magnesium stearate were obtained from FMC Corporation (United States). Aspartame was obtained from (Lubon Industry Co., Ltd, China). Potassium dihydrogen Phosphate, hydrochloric acid, and sodium hydroxide were procured from Merck (Germany).

## Preformulation studies by SeDeM-ODT expert tool

**Fig 1:**
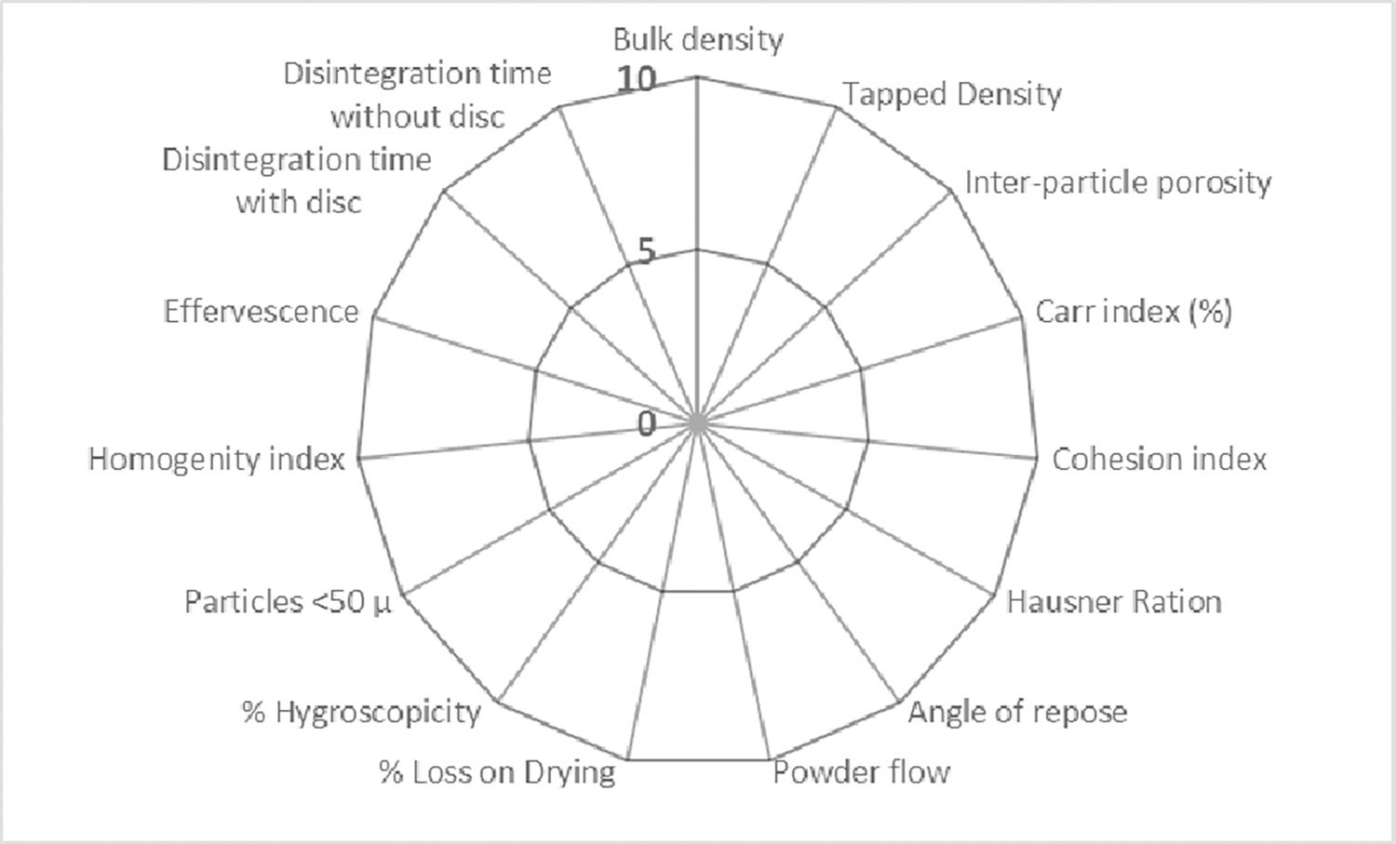
Radar diagram of SeDeM expert ODT.

Flurbiprofen was estimated for its suitability for the direct compression method by the SeDeM diagram expert tool. Therefore, API and ludipress were calculated with five incidences (dimension, compressibility, flowability/powder flow, lubricity/stability and lubricity/dosage). While flurbiprofen—ODT powder blends were calculated by the SeDeM–ODT tool. This tool has five similar incidences as in the SeDeM diagram expert tool but it has a new incidence (disgregability) derived from 15 parameters (S1 Table in S1 File) (Fig 1) [8]. These parameters were performed using different compendial methods and non-compendial methods. All the tests were carried out in triplicate to avoid the chances of variation [9]. The detailed methodology is explained in the supplementary section (SeDeM Methodology in S1 File) [8–12].

### Thermal gravimetric analysis (TGA) and Differential scanning calorimetry (DSC)

The thermal stability of the optimized formulation was evaluated by employing SDT 650 simultaneous TGA-DSC (thermal gravimetric analysis—differential scanning calorimetry thermal analyzer) [13]. Approximately, 4 mg of the sample was placed in the holding pan and an empty platinum holder was used as a reference. Dynamic scanning calorimetry and thermogravimetric analysis were performed on the sample at a rate of 10°C/min and the flow rate of nitrogen gas was maintained at 99.98ml/min.

### Scanning Electron Microscopy (SEM)

The SEM analysis of flurbiprofen (API) was performed to determine the surface morphology using a scanning electron microscope (JEOL JSM 6380, Japan) at 25 -30kV. The sample was prepared and placed on aluminium studs using double-sided adhesive carbon type which was sputter and gold coated (250 0A JFC– 1500 JEOL). SEM photograph was examined [13].

## Design of Experiments (DoE) for flurbiprofen orally disintegrating tablet

Nine different formulations of flurbiprofen ODTs were developed by central composite design (CCD) using Design Expert^®^ 11.0 software (Stat-Ease, Inc, Minneapolis). As CCD gives an accurate way to analyze statistically two factors at three levels. The independent variables were ludipress (X_1_) (49–55%) and croscarmellose sodium (X_2_) (1–5%) which were established at low (-1) and high (+1) levels. Excipient such as aspartame was used at a fixed concentration (1%). Also, lubricants were used i.e., talc (2.36%), magnesium stearate (1%), and aerosol 200 (0.14%) (standardized formula) [11]. Hardness, friability, and disintegration tests were selected as responses. To estimate the reliability of the findings, an analysis of variance (ANOVA) was performed. Graphical demonstration was carried out using a response surface plot to determine the influence of each factor on the response. The model was considered significant at a 5% level of significance (p ≤ 0.05). The model equation was established for each critical response [8].

### Compression of powder blends

Powder blends were compressed using the direct compression method. The calculated quantity of all the formulation ingredients was weighed accurately and passed through 20 mesh sieves separately and mixed for optimized mixing time i.e., six minutes by tumbling properly in a polybag. Talc, magnesium stearate, and aerosil 200 were added and mixed for a further three minutes. Powder blends were compressed using a single punch machine (Korch, Frankfurt, Germany).

### Quality evaluations of orally disintegrating tablets of flurbiprofen formulations (F1-F9)

The quality evaluations of all the formulations were carried out using various tests. Randomly 20 tablets from each formulation were individually taken and their weight variation test was performed with the help of digital top-load balance (Sartorius, Germany). To assess the crushing strength of the tablets, random (n = 20) tablets were selected. Tablets were assessed by Pfizer hardness tester. The thickness and diameter tests of the tablets (n = 20) were performed by a digital vernier calliper (Seiko, China). The % friability was determined by Roche type friability tester (Erweka D2800, Heusenstamm, Germany). The wetting time of the formulations were also performed by placing single tablet on a previously soaked filter paper and the determine the time for complete wetting of tablet [14].

### Disintegration test

For the disintegration test, a single tablet from each formulation is placed in a beaker having 5 mL of simulated saliva fluid (SSF) with pH 6.8 at 37 ± 0.5˚C. The time for tablet disintegration is noted for each tablet [15]. Khan et al. optimized atenolol granules by high shear granulation method. Delayed disintegration time was observed due to poor water penetration into the tablet core and they observed the disintegration time in mins [14]. Khan et al. formulated ODTs of glimepiride, the disintegration time observed was less than 60 seconds [7].

### Assay

For assay determination, twenty tablets from each formulation (F1 –F9) were weighed and crushed. The amount was just equal to the weight of the tablet i.e., the average was mixed with sodium hydroxide and then diluted to prepare 0.001% concentration and determine the assay at 247 nm using UV-visible spectrophotometer (UV1800 Shimadzu Corporation Kyoto, Japan) [5].

### Single and multiple point dissolution assessment

The single and multiple-point dissolution tests of all formulations were conducted using 900 ml of phosphate buffer pH 7.2. Multiple point dissolution tests were performed at various time intervals i.e., 2, 4, 6, 8, 10, 12, and 15 min. Both tests were performed using dissolution instrument (II) at 50 rpm at 37 ± 0.5°C. The samples of 5ml were withdrawn at predetermined time points. To maintain sink condition, the medium was substituted by fresh buffer. In addition, the samples were strained by a millipore filter of 0.45μm pore size to eliminate any remaining particles. The results were estimated using a UV- UV-visible spectrophotometer (UV-1800 Shimadzu Corporation Kyoto, Japan) at a wavelength λmax of ∼247 nm [5].

### Analysis of release kinetic

Dissolution data of formulations (F1 –F9) were subjected to various kinetic models using DD-Solver [16] like First–Order ($In\;Q_{t}=In\;Q_{0}-k_{1}t$) [Eq 1] [17], Higuchi ${{(Q}_{t}=K}_{H}t\frac{1}{2})$ [Eq 2] [18], Hixson–Crowell cube root law $\left(Q_{0}^{1/3}-Q_{t}^{1/3}=K_{HC}\times t\right)$ [Eq 3] [19] and Weibull model (Log[−ln(l−m)]=blog(t−Ti)−logα) [Eq 4] [20].

Where;

*Q*_*t*_ is the amount of drug released at time *t*, *Q*_0_ is the initial quantity of the drug in the formulation and release rate constants for the First order, Higuchi, Hixon Crowell, and Weibull models are *k*_1_, *k*_*H*_ *and k*_*HC*_ respectively. These models were evaluated by DD-solver an add-in program for Microsoft Excel ^TM^ 2010 (Microsoft Corporation, USA).

### Stability studies

Stability studies were carried out on all formulations at accelerated temperatures i.e. 40 + 2°C and 75 ± 5% RH (NuAire, Plymouth, MN, United States) for six months Various tests have been performed to assess the physical features i.e. assay and *in–vitro* dissolution test. The shelf life of different formulations was measured using Minitab® (17.0) software (Minitab, Pennsylvania, United States).

## Result and discussion

### SeDeM based characterization of flurbiprofen and ludipress

SeDeM expert system does not create profiles of various excipients but its goal is to indicate whether a precise excipient is appropriate for the direct compression method or not. If the radius value is < 5, it will show an insufficiency in that specific area. This system predicts the desired amount of the excipient to rectify the API inadequacy.

**Fig 2:**
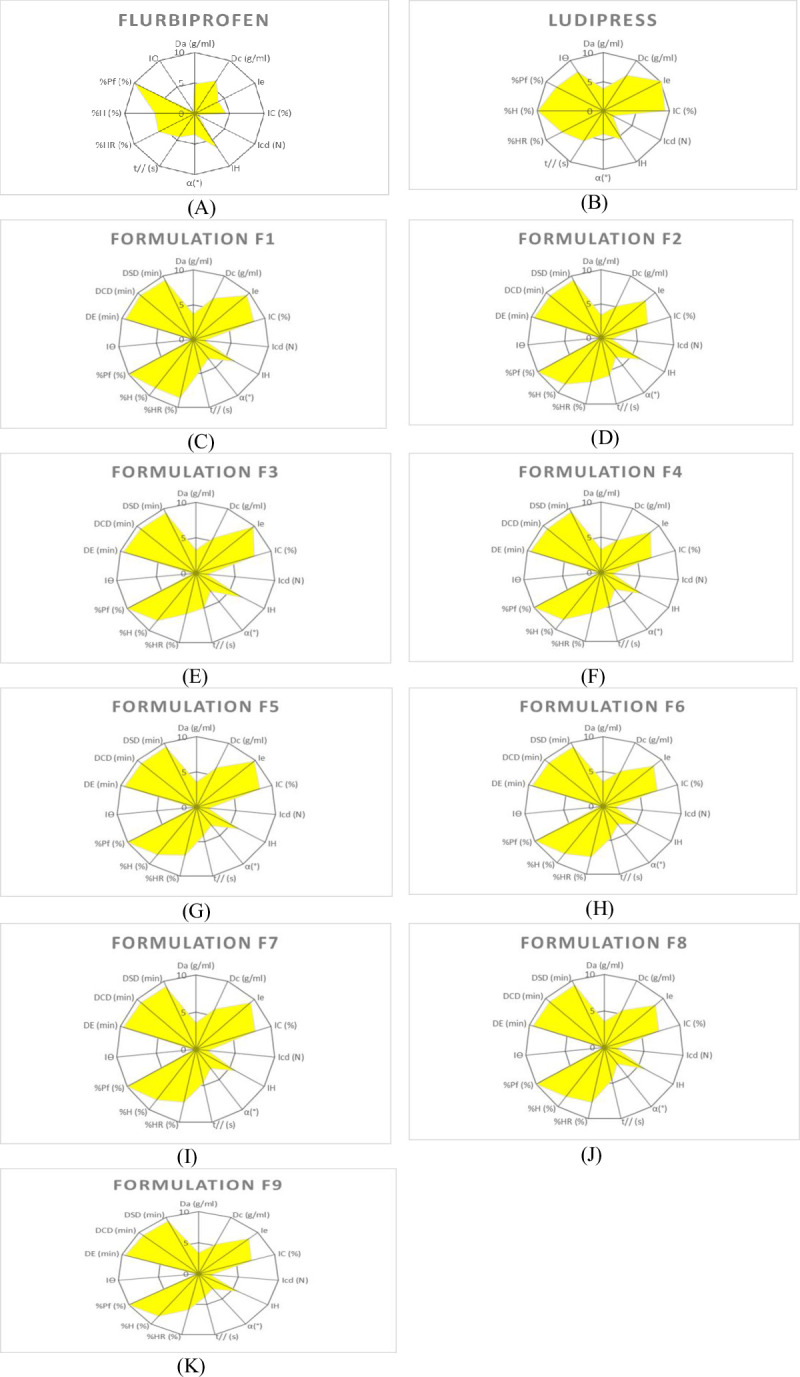
SeDeM diagram (Radar Plot) of ludipress, flurbiprofen and formulations (F1-F9). (A) Flurbiprofen (API) (B) Ludipress (Superdisintegrant) (C) Formulation F1 (D) Formulation F2 (E) Formulation F3 (F) Formulation F4 (G) Formulation F5 (H) Formulation F6 (I) Formulation F7 (J) Formulation F8 (K) Formulation F9.

Before preparing powder blends of formulations, API and binder were estimated for their appropriateness for the direct compression method using the SeDeM expert system. So flurbiprofen and ludipress were subjected to 12 parametric tests to obtain the radius values that were used to get *the polygons (Fig 2). The experimental values of parameters/indices, mean incidence factors and* indices of flurbiprofen and ludipress are shown (Table 1). The results of the SeDeM diagram indicated that the ludipress is suitable for direct compression excipient and possesses a high value of 6.40 for the “Good Compressibility Index” (IGC) as compared to flurbiprofen having an IGC value of 4.50. For “Index Parameter” (IP) and “Parameter Profile Index (IPP) values for flurbiprofen were 0.42 and 4.73 and for binder were 0.75 and 6.72 respectively. By observing the SeDeM diagrams it was found that ludipress possess excellent coverage in various aspects of parameter indices above 5 (minimum value) so the blend of flurbiprofen and ludipress could cover the insufficiency and would be appropriate for the direct compression method. The least amount of ludipress used to overcome the lower value of deficient incidence parameter as mentioned in the supplementary section (SeDeM Methodology in S1 File), was found to be 49.1% % so the active compound value gets improved.

**Table 1:** Experimental values of parameters / indices, mean incidence factors, and indices of flurbiprofen and ludipress.

SeDeM Experimental Values for API and Excipients	Parameter / Indices (*r-value*)												Mean Incidence Factor (Ratio)					Indices			
	*Bulk Density (Da)*	*Tapped Density (Dc)*	*Interparticle Porosity (Ie)*	*Carr’s Index (IC)*	*Cohesion Index (Icd)*	*Hausner Ratio (IH)*	*The angle of Repose (θ)*	*Powder Flow (t”)*	*Loss On Drying (% HR)*	*Hygroscopicity (%H)*	*Particle Size <50 (%Pf)*	*Homogeneity Index (Iθ)*	*Dimension*	*Compressibility*	*Flowability/ Powder Flow*	*Lubricity/Stability*	*Lubricity/Dosage*	*Index parameter (IP)*	*Parameter Profile Index (IPP)*	Index of Good Compressibility (IGC)**	Remarks*
**Flurbiprofen**	**4.80**	**6.20**	**3.92**	**4.52**	**0.56**	**6.55**	**3.49**	**4.50**	**6.00**	**5.70**	**9.99**	**0.49**	**5.50**	**3.00**	**4.85**	**5.85**	**5.24**	**0.42**	**4.73**	**4.50**	A*
**Ludipress**	**3.80**	**7.10**	**10.19**	**9.30**	**1.71**	**5.66**	**3.96**	**5.95**	**7.23**	**9.90**	**8.18**	**7.71**	**5.45**	**7.07**	**5.19**	**8.57**	**7.95**	**0.75**	**6.72**	**6.40**	***A***

### Design of Experiments (DoE) For the development of flurbiprofen–ODT tablets

DoE is an important statistical tool which had been used by the pharmaceutical industry for different stages of product manufacturing due to good yield, minimum number of experiments, and reduced manufacturing cost as well [21].

To obtain significant pharmaceutical features, the response surface methodology (RSM) technique has been widely utilized to generate data for tablet formulation. The central composite design has been extensively reported DoE methodology for the optimization of orally disintegrating tablets [22]. Bushra et al. formulated and optimized aceclofenac tablets by central composite rotatable design. The response factors were shown to be significant as proposed by RSM. Tablets of aceclofenac were prepared by the direct compression technique and optimized by CCRD using three input variables having five distinct levels [23]. In this study, nine different formulations were developed using two independent variables and three ludipress (X1) (49–55%) and Ac-Di-Sol (0.1–5%) with five different input factors levels while hardness (Y_1_), disintegration time (Y_2_) and % friability (Y_3_) were selected as responses (Table 2 and S2 Table in S1 File). Response surface methodology (RSM) has been adopted for the development of clobazam orally disintegrating tablets [24]. Remya et al. performed a comparative study to determine the effects of super-disintegrants for the development of cefixime oral disintegrating tablets. Formulations with croscarmellose sodium showed excellent disintegration results [25]. Mor et al. also studied different super-disintegrants for the development of lornoxicam fast-dissolving tablets [26].

**Table 2:** Composition of flurbiprofen-ODT formulations (F1-F9).

Formulations	Ludipress	Croscarmellose Sodium	Aerosil 200	Talc	Aspartame	Magnesium Stearate	API	Tablet Weight
	% (mg)	% (mg)	% (mg)	% (mg)	% (mg)	% (mg)	Mg	Mg
**F1**	49 (107.692)	1 (2.197)	0.14 (0.307)	2.36 (5.186)	1 (2.197)	1 (2.197)	100	219.780
**F2**	56.242 (155.120)	3 (8.274)	0.14 (0.386)	2.36 (6.509)	1 (2.758)	1 (2.758)		275.805
**F3**	49 (118.072)	5 (12.048)	0.14 (0.337)	2.36 (5.686)	1 (2.409)	1 (2.409)		240.963
**F4**	52 (128.395)	3 (7.407)	0.14 (0.345)	2.36 (5.827)	1 (2.469)	1 (2.469)		246.913
**F5**	52 (138.004)	5.82 (15.445)	0.14 (0.371)	2.36 (6.263)	1 (2.653)	1 (2.653)		265.392
**F6**	47.75 (106.703)	3 (6.703)	0.14 (0.312)	2.36 (5.273)	1 (2.234)	1 (2.234)		223.463
**F7**	52 (120.012)	0.171 (0.394)	0.14 (0.323)	2.36 (5.446)	1 (2.307)	1 (2.307)		230.792
**F8**	55 (139.240)	1 (2.531)	0.14 (0.354)	2.36 (5.974)	1 (2.531)	1 (2.531)		253.164
**F9**	55 (154.929)	5 (14.084)	0.14 (0.394)	2.36 (6.647)	1 (2.816)	1 (2.816)		281.690

### SeDeM–ODT based analysis of formulation blends

Central composite design yielded a total of nine formulations (Table 2). The proposed formulation blends (F1 –F9) were then subjected to the SeDeM–ODT expert system for the suitability of the direct compression method before adding the standard mixture of talc, magnesium stearate, and aerosil 200 to see whether the powder blends on their own was appropriate enough for direct compression. The results of the SeDeM–ODT experiments showed that all the powder blends (F1 –F9) were appropriate for the direct compression technique since all the blends had IGC and IGCB values above 5 as indicated in Table 3 and Fig 2. The Index parameter (IP) and Parameter Profile Index (IPP) for all the formulation powder blends were 0.53–0.60 and 6.05–6.64 respectively. For ludipress, the lubricity/dosage values were well above the acceptable limit. Gülbağ et al., developed memantine disintegrating tablets using three different super disintegrants to enhance the compressibility of active compounds following SeDeM diagrams. They suggested that ludipress improves the compressibility of the active ingredients and found that ludipress has shown the values of lubricity/dosage within the given range [27]. Bashir et al. prepared and optimized ribavirin granules with excipients. The characteristic parametric indices were calculated and high IGCB values were observed. The powder blend had a poor flow and compressibility while the disintegration time was good due to the high water solubility [28].

**Table 3:** Experimental values of parameters / indices, mean incidence factors and indices of (F1 –F9).

Formulations	Parameter / Indices (*r-value*)															Mean Incidence Factor (Ratio)						*Indices*				
																						*Index parameter* *(IP)*	*Parameter* *Profile* *Index* *(IPP)*	*Index of Good Compressibility* (IGC)**	*Index of Good Compressibility* *&* Bucco-dispersibility (IGCB)**	Remarks*
	*Bulk Density (Da)*	*Tapped Density (Dc)*	*Interparticle Porosity (Ie)*	*Carr’s Index (IC)*	*Cohesion Index (Icd)*	*Hausner Ratio (IH)*	*Angle of Repose (θ)*	*Powder Flow (t”)*	*Loss On Drying (% HR)*	*Hygroscopicity (%H)*	*Particle Size <50 (%Pf)*	*Homogeneity Index (Iθ)*	*Effervescence*	*Disintegration Time with Disc (DCD)*	*Disintegration Time without Disc (DSD)*	*Dimension*	*Compressibility*	*Flowability/ Powder Flow*	*Lubricity/Stability*	*Lubricity/Dosage*	*Disgregability*					
**F1**	**3.70**	**6.46**	**9.50**	**8.44**	**1.51**	**6.30**	**3.35**	**4.50**	**8.64**	**8.60**	**9.99**	**0.32**	**9.54**	**9.45**	**9.32**	**5.08**	**6.48**	**4.72**	**8.62**	**5.16**	**9.39**	**5.08**	**0.67**	**6.64**	**6.31**	*A*
**F2**	**3.40**	**5.10**	**8.16**	**6.66**	**1.49**	**6.38**	**3.49**	**5.67**	**6.60**	**8.46**	**9.99**	**0.34**	**9.56**	**9.32**	**9.27**	**4.25**	**5.44**	**5.18**	**7.53**	**5.17**	**9.30**	**4.25**	**0.73**	**6.26**	**5.95**	*A*
**F3**	**3.31**	**5.41**	**9.82**	**7.78**	**1.52**	**6.81**	**3.17**	**4.99**	**5.89**	**8.28**	**9.99**	**0.41**	**9.56**	**9.38**	**9.32**	**4.36**	**6.37**	**4.99**	**7.09**	**5.20**	**9.35**	**4.36**	**0.67**	**6.38**	**6.06**	*A*
**F4**	**3.30**	**5.00**	**8.58**	**6.80**	**1.52**	**6.07**	**2.98**	**4.83**	**5.88**	**8.28**	**9.99**	**0.44**	**9.60**	**9.27**	**9.45**	**4.15**	**5.63**	**4.63**	**7.08**	**5.22**	**9.36**	**4.15**	**0.67**	**6.13**	**5.83**	*A*
**F5**	**3.58**	**6.18**	**9.79**	**8.41**	**1.55**	**6.14**	**3.20**	**4.16**	**6.95**	**8.33**	**9.99**	**0.34**	**9.50**	**9.32**	**9.38**	**4.88**	**6.58**	**4.50**	**7.64**	**5.17**	**9.35**	**4.88**	**0.67**	**6.45**	**6.13**	*A*
**F6**	**3.50**	**5.50**	**8.65**	**7.27**	**1.56**	**5.16**	**3.20**	**4.66**	**7.43**	**8.42**	**9.98**	**0.45**	**9.48**	**9.32**	**9.32**	**4.50**	**5.83**	**4.34**	**7.93**	**5.22**	**9.32**	**4.50**	**0.67**	**6.26**	**5.95**	*A*
**F7**	**3.54**	**5.88**	**9.36**	**7.95**	**1.53**	**5.80**	**3.19**	**4.61**	**7.23**	**8.40**	**9.99**	**0.27**	**9.56**	**9.38**	**9.22**	**4.71**	**6.28**	**4.53**	**7.82**	**5.13**	**9.30**	**4.71**	**0.67**	**6.39**	**6.07**	*A*
**F8**	**3.50**	**5.50**	**8.65**	**7.27**	**1.55**	**5.98**	**2.76**	**4.54**	**7.70**	**8.31**	**9.99**	**0.30**	**9.53**	**9.27**	**9.32**	**4.50**	**5.82**	**4.43**	**8.01**	**5.15**	**9.30**	**4.50**	**0.67**	**6.28**	**5.96**	*A*
**F9**	**3.40**	**5.20**	**8.48**	**6.92**	**1.57**	**5.63**	**2.99**	**3.83**	**5.89**	**8.41**	**9.99**	**0.28**	**9.56**	**9.27**	**9.32**	**4.30**	**5.66**	**4.15**	**7.15**	**5.14**	**9.30**	**4.30**	**0.67**	**6.05**	**5.75**	*A*

### TGA-DSC

**Fig 3:**
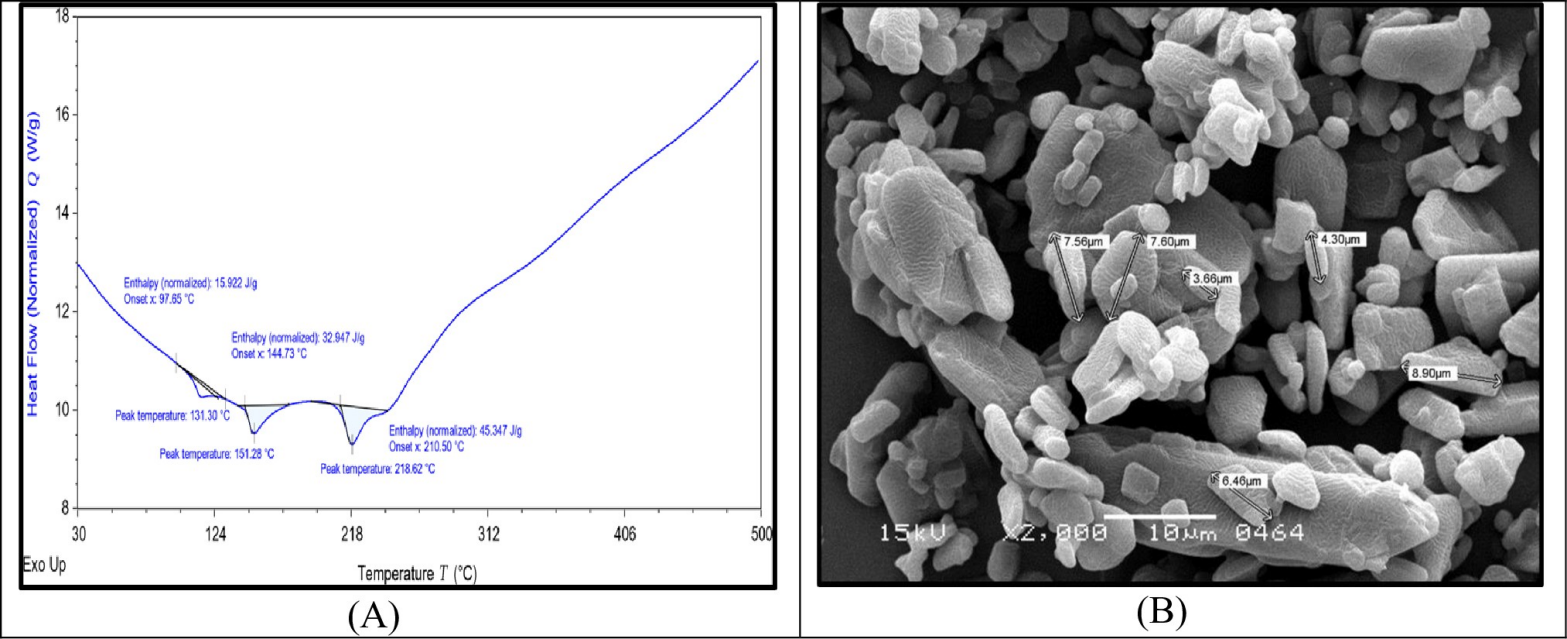
Thermal stability and surface morphology of sample and API respectively. (A) Thermal gravimetric analysis (TGA) and Differential scanning calorimetry (DSC) of Optimized Flurbiprofen–ODT Formulation. (B) SEM analysis of flurbiprofen (API).

In this article flurbiprofen–ODT formulation was examined by TDA-DSC for thermal decomposition. The thermogram demonstrated a spike at melting point near 218.62°C for formulation. Degradation was indicated by an endothermic spike by the thermal flow curve which appeared at 97.65–210.50°C for formulation (Fig 3).

### SEM analysis

In this research, the SEM analysis was carried out on flurbiprofen. Results indicated some rod-shaped or irregular particles having rough surfaces. Pictorial analysis at x2000 magnification showed some aggregated, large particles having irregular surfaces. The particle sizes were ranged from 3.66 –to 8.90μm (Fig 3).

### RSM plot and ANOVA summary

**Fig 4:**
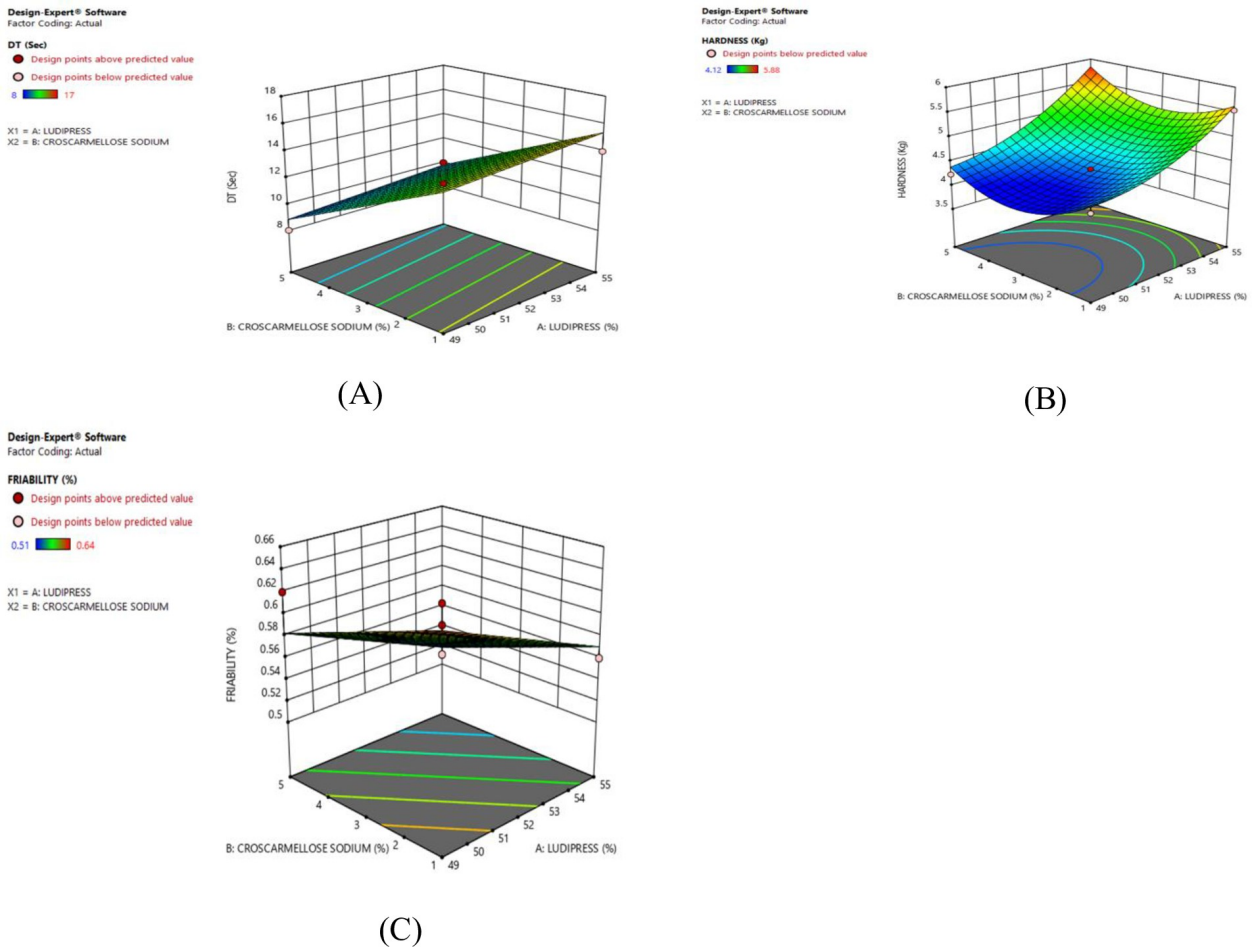
Response surface plots of flurbiprofen–ODT tablets (F1 –F9) indicating the effect of independent variables. (A) Disintegration Time (B) Hardness and, (C) Friability.

**Fig 5:**
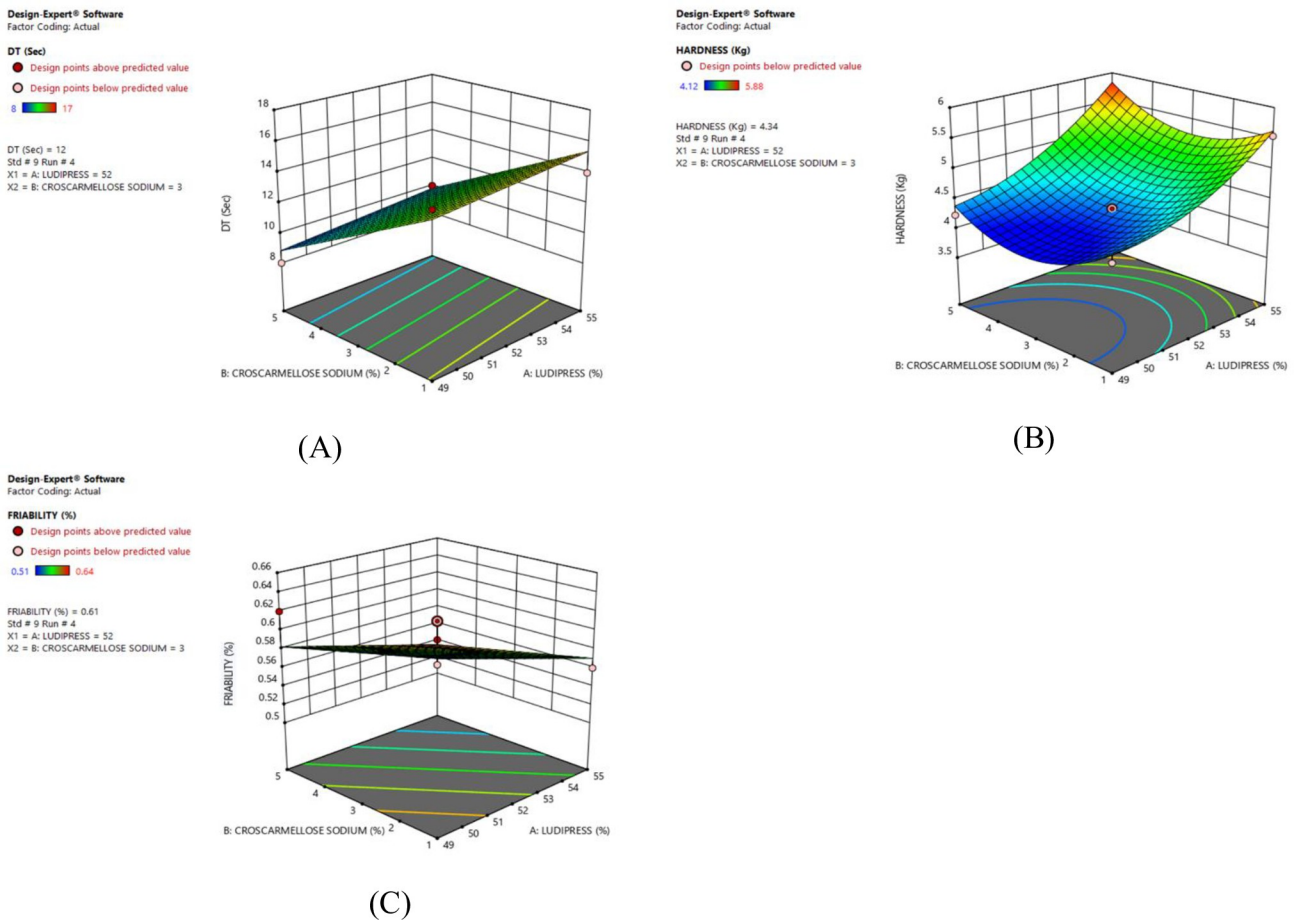
Response surface plots of optimized flurbiprofen–ODT tablets (F4) indicating the effect of independent variables. (A) Disintegration time (B) Hardness and, (C) Friability.

It is shown in the RSM plot that with the increase in the concentration of ludipress the disintegration time for tablets is high and vice versa. The RSM plots for disintegration time for F1 –F9 and F4 (Figs 4 and 5) respectively. The ANOVA summary for the disintegration time indicated that the model F-value of 39.89 implies the model is significant. The probability value was found to be less than 0.05 showing that the linear model was significant. The “Adeq precision” was 15.246 indicating an adequate signal. Linear model can be used to navigate the design space (S4 Table in S1 File). The RSM plots for hardness for F1 –F9 and F4 (Figs 4 and 5) showed that hardness was high as the concentration of binder (ludipress) was increased. The F value, Adeq precision, and probability were found to be 19.82, 11.346, and < 0.05 respectively indicating the linear and quadratic model was significant (S5 Table in S1 File). The RSM plot for % friability for F1 –F9 and F4 as shown in Figs 4 and 5 respectively indicated that the % friability was decreased as the concentration of ludipress was high. The F value, Adeq precision, and probability were found to be 5.49, 5.73and < 0.05 respectively showing that the linear model was acceptable with the adequate signal (S6 Table in S1 File). The probability value and coded equations of selected responses were presented in (S3 Table in S1 File).

### Evaluation of quality attributes of formulations

The formulated tablets of flurbiprofen were evaluated by various physicochemical evaluations. Subsequently, all the formulations (F1 –F9) were formulated through a systematic approach and showed good physicochemical attributes. The weight variation for all the formulated tablets was found satisfactory. The mean weight of all the formulations was found within the USP limit of ± 7.5% of the given weight. Similarly, the thickness and hardness were found to be inadequate range. The % friability for all formulations was < 1%. The disintegration time for F1 –F9 was observed to be in the range of 10–13 sec. Sipos et al. formulated ibuprofen containing ODTs by using the SeDeM expert system technique. The disintegration time was observed within the stipulated limit of 3 minutes and less than 80% of ibuprofen was dissolved after 60 minutes according to the USP monograph [29]. Fouad et al. developed diacerein ODTs. They performed disintegration test and the results were observed in secs while maximum % drug release were obtained in 60 min [15]. The wetting time of all formulations were in the range of 8 ± 3.18–13 ± 1.58 sec indicating the time taken for the penetration of the medium in the tablet core [14]. Finally, the results of the assay and single-point dissolution test suggested that all the formulations (F1 –F9) were found to be within acceptable limits. Thus, all formulations satisfy various parameters of quality attributes (Table 4). The optimized formulation was found to be F4 as it showed rapid disintegration with excellent dissolution and assay results.

**Table 4:** Physicochemical quality attributes of flurbiprofen–ODT formulations (F1- F9).

*Formulations*	*Weight Variation* *(mg)*	*Thickness* *Variation* *(mm)*	*Hardness* *Variation* *(kg)*	*Friability Test* *(%)*	*Disintegration* *Time* *(sec) and)*	*Dissolution* *Test* *(%)*	*Assay* *Test* *(%)*	*Wetting Time (sec)*
*F1*	219.9 ± 2.231	4.29 ± 006	3.09 ± 0.08	0.56	11	98 .00 ± 1.17	99.30 ± 0.92	9 ± 2.32
*F2*	275.8 ± 2.567	4.36 ± 0.07	3.16 ± 0.06	0.52	12	99.92 ± 0.37	99.01 ± 0.54	13 ± 1.58
*F3*	240.9 ± 2.560	4.75 ± 0.04	3.10 ± 0.09	0.54	10	98.65 ± 0.42	98.68 ± 0.57	12 ± 3.56
*F4*	246.9 ± 2.277	4.18 ± 0.02	3.05 ± 0.08	0.53	13	990.66 ± 0.75	98.48 ± 1.11	9 ± 4.26
*F5*	265.3 ± 1.593	4.59 ± 0.01	3.17 ± 0.05	0.51	12	99.23 ± 0.47	98.84 ± 0.28	8 ± 3.18
*F6*	223.4 ± 2.583	4.95 ± 0.07	3.18 ± 0.06	0.53	12	99.26 ± 0.35	98.56 ± 0.52	10 ± 4.17
*F7*	230.7 ± 2.922	4.62 ± 0.04	3.12 ± 0.07	0.55	11	98.06 ± 0.39	98.39 ± 0.09	8 ± 1.55
*F8*	253.1 ± 2.573	4.10 ± 0.04	3.01 ± 0.05	0.58	13	99.03 ± 1.21	98.40 ± 0.55	11 ± 4.65
*F9*	281.6 ± 2.927	4.81 ± 0.06	3.21 ± 0.05	0.57	13	98.36 ± 0.45	99.00 ± 0.91	10 ± 5.12

### Analysis of multiple point dissolution studies

**Fig 6:**
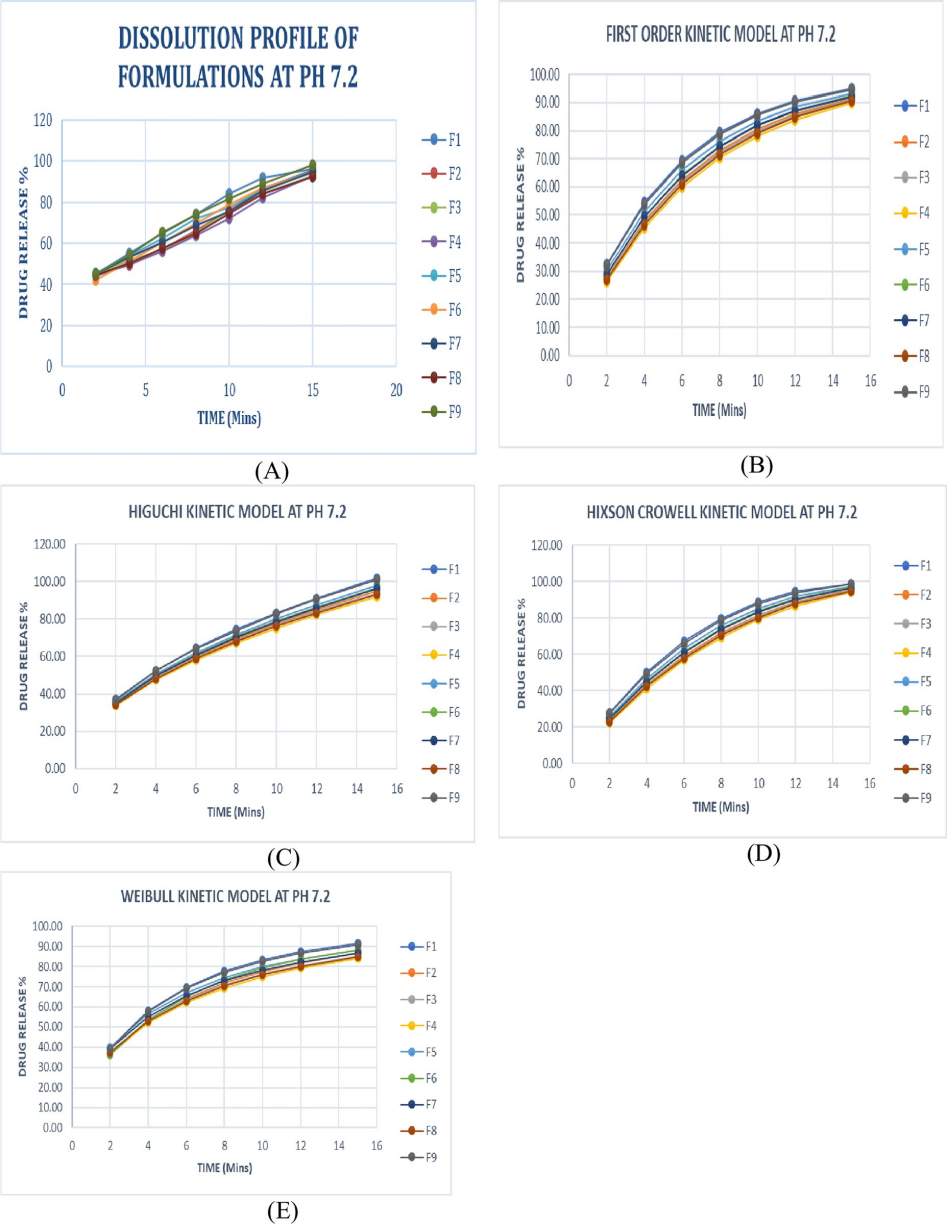
Graphical representation of *in vitro* release pattern and release kinetics model of formulations F1- F9 at 7.2. (A) *Invitro* Drug release pattern for formulations (F1- F9). (B) First Order, Higuchi, Hixon Crowell, and Weibull Model of formulations (F1- F9).

The most significant aspect for absorption of conventional solid dosage form depends on the factor on the release of the drug, dissolution of the drug in a biological environment, and absorptivity through GIT. *In-vitro* dissolution evaluation gives an estimate for *in-vivo* dissolution performance [20]. In the current study, the *in–vitro* dissolution studies of newly developed and optimized formulations were carried out at a dissolution medium pH 7.2 (Fig 6). All the formulations followed the Higuchi kinetic model at pH 7.2 with excellent *r*^2^ values which were found to be 0.923–0.972 (Table 5). Graphical presentation of the first order [Eq 1], Higuchi [Eq 2], Hixon Crowell [Eq 3], and Weibull [Eq 4] of F1-F9 at pH 7.2 were mentioned (Fig 6). Yasmin et al. in 2020 determined that optimized aceclofenac dispersible tablets showed the highest *r*^2^ value at phosphate buffer pH 6.8 and all the formulations followed the Weibull model [20].

**Table 5:** Release kinetics of formulations (F1-F9) at pH 7.2.

	FORMULATIONS								
MODELS	F1	F2	F3	F4	F5	F6	F7	F8	F9
	**FIRST ORDER**								
***r*** ^**2**^	0.902	0.812	0.774	0.764	0.833	0.891	0.797	0.781	0.891
***k1(h-1)***	0.198	0.163	0.160	0.152	0.180	0.171	0.171	0.157	0.193
* *	**HIGUCHI MODEL**								
***r*** ^***2***^	0.953	0.940	0.923	0.925	0.936	0.972	0.930	0.929	0.961
***kH(h-1/2)***	26.301	24.559	24.207	23.755	25.312	24.940	24.813	24.054	26.116
* *	**HIXON CROWELL MODEL**								
***r*** ^***2***^	0.819	0.740	0.662	0.659	0.708	0.810	0.674	0.671	0.802
***kHC(h-1/3)***	0.052	0.043	0.042	0.041	0.047	0.045	0.045	0.042	0.051
* *	**WEIBULL MODEL**								
***r*** ^***2***^	0.944	0.876	0.887	0.879	0.929	0.941	0.908	0.892	0.939
***β***	0.792	0.748	0.693	0.691	0.715	0.776	0.699	0.695	0.780
***α***	3.456	3.748	3.425	3.553	3.240	3.806	3.281	3.487	3.440

### Stability studies

**Fig 7:**
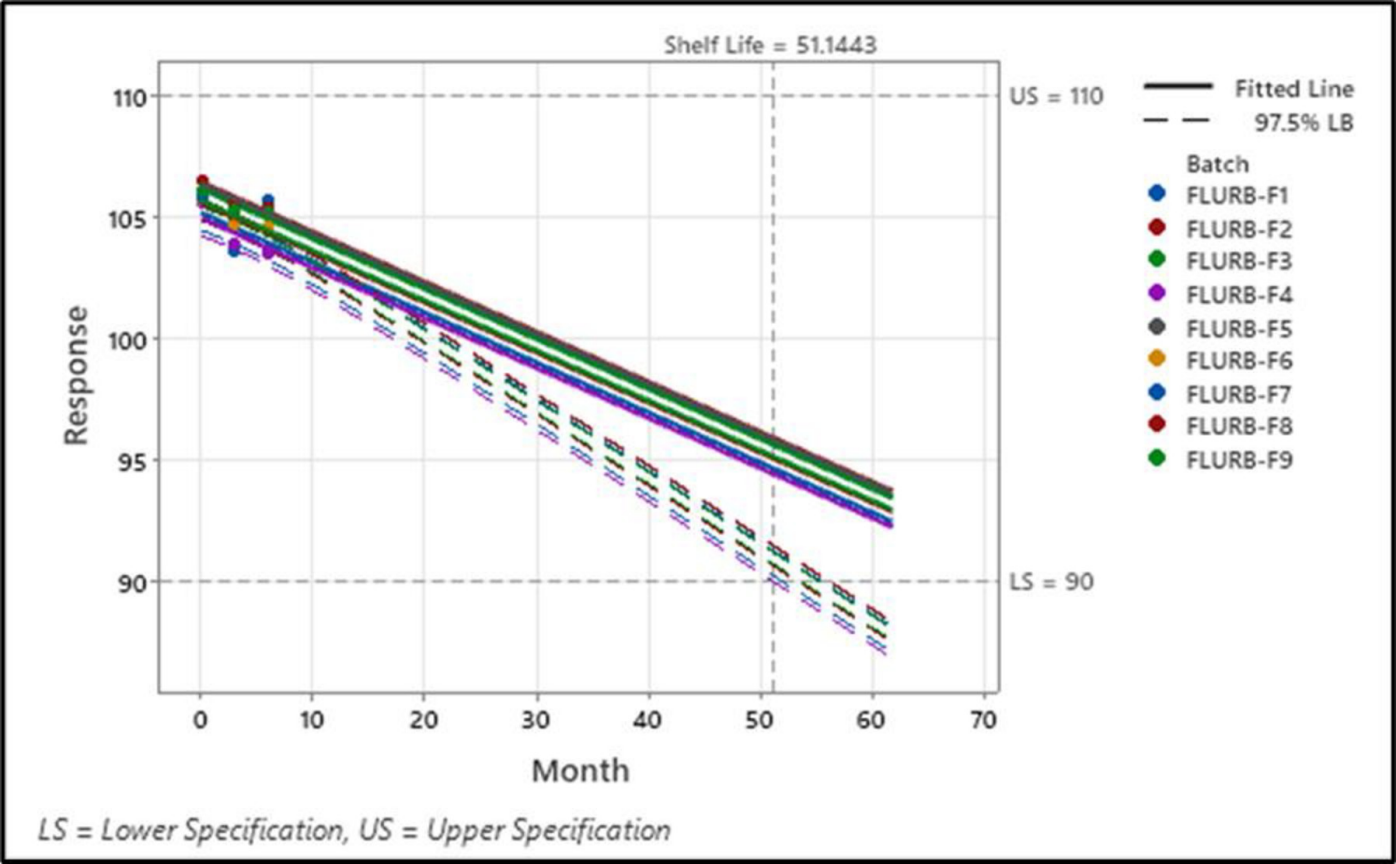
Shelf-life estimation of F1 –F9 at accelerated state.

Stability studies aim to determine the shelf life where the drug product is placed for a certain period under varying conditions of relative humidity (RH) and temperature. For accelerated conditions, all the formulations were placed at 40 ± 5°C and 75 ± 5% RH. Various quality was performed tests including assay, dissolution, and disintegration tests during 0, 3, and 6 months studies. All the formulations expressed good quality characteristics, % release of the drug at 0 months was 98.126 ± 0.974 to 99.663 ± 0.697 at 3 months 98.004 ± 0.431 to 99.082 ± 0.821 and at 6 months it is reduced 97.010 ± 0.221 to 99.003 ± 0.084, for assay % at 0 months 98.452 + 0.258 to 99.634 + 0.189, at 3 months 98.008 ± 0.824 to 99.210 ± 0.741, and at 6 months 97.922 ± 0.428 to 99.194 ± 0.421, and disintegration time at 0 months was recorded 10 to 13 secs, at 3 months increase in disintegration time was recorded 11 to 14 secs, and for 6 months further increase in disintegration time was recorded 12 to 15 secs and disintegration time results. Results indicated that all the formulations were found stable (Table 6). Shelf life was also determined and it was found to be in the range of 51.144–56.186 months (Fig 7) and (S7 and S8 Tables and S1–S9 Figs in S1 File).

**Table 6:** Stability studies of flurbiprofen–ODT formulations at accelerated conditions.

*Formulations*	*0 Month*			*3 Month*			*6 Month*		
	*Assay* *(%)* *n = 3*	*Dissolution* *(%)* *n = 6*	*Disintegration time* *(sec)* *n = 3*	*Assay* *(%)* *n = 3*	*Dissolution* *(%)* *n = 6*	*Disintegration* *Time* *(sec)* *n = 3*	*Assay* *(%)* *n = 3*	*Dissolution* *(%)* *n = 6*	*Disintegration* *Time* *(sec)* *n = 3*
*F1*	99.30 ± 0.26	98.45 ± 0.27	10 ± 0.16	99.21 ± 0.74	99.00 ± 0.08	11 ± 0.01	99.19 ± 0.42	99.00 ± 0.08	12 ± 0.17
*F2*	99.12 ± 0.41	98.88 ± 0.24	12 ± 0.10	98.20 ±0.84	99.08 ± 0.82	13 ± 0.01	98.10 ± 0.44	98.90 ± 0.05	14 ± 0.07
*F3*	99.03 ± 0.89	99.36 ± 0.85	11 ± 0.02	98.02 ± 0.44	98.34 ± 0.25	12 ± 0.10	98.01 ± 0.43	98.20 ± 0.25	13 ± 0.13
*F4*	98.48 ± 0.56	99.66 ± 0.69	13 ± 0.01	98.10 ± 0.64	98.51 ± 0.62	14 ± 0.01	98.02 ± 0.22	97.01 ± 0.22	15 ± 0.02
*F5*	98.45 ± 0.25	98.46 ± 0.49	12 ± 0.06	98.10 ± 0.38	98.66 ±0.01	13 ± 0.02	97.92 ± 0.42	98.02 ± 0.85	14 ± 0.17
*F6*	99.63 ± 0.18	99.36 ± 0.89	12 ± 0.05	98.30 ± 0.48	98.00 ± 0.43	13 ± 0.06	98.01 ± 0.53	97.02 ± 0.25	14 ± 0.12
*F7*	98.46 ± 0.37	98.12 ± 0.97	11 ± 0.09	98.01 ± 0.82	98.50 ± 0.75	12 ± 0.02	97.99 ± 0.56	97.88 ± 0.28	13 ± 0.22
*F8*	99.62 ± 0.25	99.36 ± 0.49	13 ± 0.01	98.01 ± 0.56	98.35 ± 0.52	14 ± 0.13	98.01 ± 0.43	97.48 ± 0.10	15 ± 0.24
*F9*	98.33 + 0.45	98.39 + 0.74	13 ± 0.01	99.01 ± 0.72	99.00 ± 0.12	14 ± 0.01	98.23 ± 0.73	98.02 ± 0.52	15 ± 0.01

## Conclusion

Based on these results, the SeDeM–ODT diagram is a way to design Flurbiprofen–ODT tablets by cost-effective direct compression method. The central composite design was successfully applied on the Flurbiprofen–ODT tablets. Formulations were examined on various quality tests. Hence selecting suitable excipients with appropriate concentration is helpful to ensure a palatable and stable dosage form for the patient.

## Supporting information

S1 File Raw data. (DOCX)
