# Supplementary material for: Development and characterisation of orally disintegrating flurbiprofen tablets using SeDeM-ODT tool
Source: PLoS One. 2024 Oct 29;19(10):e0309894. doi: 10.1371/journal.pone.0309894 (PMC11521243; doi:10.1371/journal.pone.0309894)
Supplement: S1 File — (DOCX) [file pone.0309894.s001.docx]

**SUPPLEMENTARY DATA FILE**

***SeDeM Methodology***

**CHARACTERIZATION OF PARAMETERS**

Fifteen SeDem ODT parameters were studied following European pharmacopoeia:

**Dimension Factor**

Parameters of dimension factor are mentioned as follows:

***Bulk Density (***$Da$***):***

The bulk density of the powder material is evaluated according to USP guidelines, using the graduated cylinder method. The volume of powder is determined by weighing and then pouring it into the graduated cylinder. 10g of the sample is weighed and poured into the graduated cylinder. The bulk density was measured by the following equation:

 **Eq 1**

Where, P= Powder weight (g), V*a* = Apparent volume of the powder (ml), and Da= Bulk Density (g/ml).

***Tapped Density (***$Dc$***):***

Tapped density of the powder substance is determined by tapping the known volume of weighed powder in a graduated cylinder and observing the volume reduction by USP guidelines. This method can be done manually or can be done by mechanical tappers. 10g of sample is weighed and poured into a graduated cylinder and tapping is done manually until no difference in volume is seen. Tapped density was measured by the following equation:

 **Eq 2**

Where, P= Powder weight (g), Vc = volume of the powder after tapping (ml), and Dc= Tapped Density (g/ml).

***COMPRESSIBILITY:***

This factor comprises parameters that are related to the compressibility of the powder substance. Parameters in this group are:

***Inter-particle Porosity (𝐼𝑒):***

Inter-particle porosity can be calculated by values of bulk density and tapped density. It was calculated by the following equation;

$Ie=\frac{Dc-Da}{Dc \times Da}$  **Eq 3**

***Carr’s Index (𝐼𝐶):***

Carr’s index can be determined based on bulk density and tapped density. It was estimated by the following equation*;*

**Eq 4**

***Cohesion Index*** $(Icd)$***:***

The crushing strength of the compressed powder is called the cohesion index. It is calculated by compression of powder and powder blends in an eccentric press machine subjected to maximum pressure and determines the average hardness of the material. The hardness values were converted from Kg into Newton (N).

***FLOWABILITY:***

This factor comprises the parameters concerning the flow ability of the powder material during compression and includes the following:

***Hausner ratio (IH):***

Hausner’s ratio is determined by the bulk density and tapped density of the powder material. It was analyzed using the following equation:

**Eq 5**

Where Hr = Hausner’s ratio, Dc tapped density of the powder (g/ml), and Da is the bulk density of the powder (g/ml).

***Angle of Repose (θ):***

This test was evaluated by the method described in European Pharmacopoeia. The procedure includes the measurement of the height and base of the material. The alpha (α) was calculated by the following equation:

**Eq 6**

Where, h= Height of powder heap (cm) and D= Diameter of powder heap (cm).

***Powder Flow (t’’):***

The powder flow is evaluated by the method described in European Pharmacopeia.40 g of powder sample was allowed to pass through an orifice and time was noted with the help of a digital stopwatch.

***LUBRICITY / STABILITY:***

Lubricity or stability during the compression of tablets is affected by the parameters included in this factor. The incidence values represent the rheological properties of the powder material.

***Loss on Drying (%HR):***

Loss on drying is evaluated gravimetrically by the USP. In this method, the powder sample was placed on a pan of moisture analyzer at 105°C for 2 hrs until the constant weight was attained and the percent loss in weight was observed.

***Hygroscopicity (% H):***

Hygroscopicity is determined by placing the weighed powder material in the humidifier chamber. The humidifier was at a relative humidity of 75% at 22.5°C for 24 hrs until the constant weight was observed.

***LUBRICITY / DOSAGE:***

Parameters that affect the lubricity and dosage of pharmaceutical formulation are included in this factor.

***Particle size under 50 µm (%Pf)***

For particle size determination sieve shaker with standard sieves of pore sizes 850, 600, 425,300, and 250 µm is used. In this test, the powder sample was placed on sieves set on the gyratory shaker and was vibrated for 10 minutes to assess the particle size below 50 µm.

***Homogeneity Index (𝐼𝜃)***

The homogeneity index is evaluated by European Pharmacopeia. The test was performed by placing powder material on different sieves (0.355mm - 0.05mm) set on a gyratory shaker for 10 minutes to evaluate particle size. The percent quantity of powder material retained on each sieve and which passes through a 50 µm sieve is assessed. The homogeneity index can be calculated by the following equation:

$I\theta=\frac{Fm}{\begin{aligned} 100+\left( dm-dm-1 \right)Fm-1+\left( dm+1-dm \right)Fm+1\left( dm-dm-2 \right)Fm-2+\left( dm+2-dm \right)Fm+2 \\ \ldots.+\left( dm-dm-n \right)Fm-n+\left( dm+n-dm \right)Fm+n \end{aligned}}$ **Eq 7**

Where,

‘F_m_’ and ‘d_m_’ = is the percentage (%) of particles in major size range and their diameter respectively.

‘n order number of particle size fraction.

‘F_m+1_’ and ‘d_m+1_’ = particles percentage (%) and diameter of the size fraction above the majority range.

‘F_m-1_’ and ‘d_m-1_’ = particles percentage (%) and diameter of the size fraction below the majority range.

***DISGREGABILITY:***

The disgregability factor comprises parameters that determine the disintegration behavior of the tablet. Parameters included are:

***Effervescence:***

The effervescence test is performed by the official monograph. The powder substance is compressed into tablets under maximum pressure. In this method, the single tablet was placed in a container having purified water at 15 – 25°C and allowed to disintegrate within 5 mins.

***Disintegration Time with Disc and with our Disc:***

The powder is compressed into a tablet under high pressure and is subjected to the disintegration time, performed on the USP disintegration apparatus. This test was performed by following the method mentioned by scientists using de-ionized water as a media held at 37 ± 2ºC temperature. [11]. The technique was comparable to the mechanical action that took place inside the mouth. The linear parametric values were calculated from the limit conversion of the values obtained (Supplementary Table S1).

**SeDeM-ODT parametric indices:**

The estimation of SeDeM-ODT parametric indices was analyzed by the following equations:

**Index Parameter (IP):**

Parametric index is the ratio of parameters having values ‘*r*’ equal to or greater than 5 to the total number of parameters determined. The parametric index is calculated by the following equation:

$IP=\frac{N^{^{\circ}}P\geq5}{N^{\circ} Pt}$ (> 0.5).  **Eq 8**

**Profile Index Parameter (IPP):**

The parameter profile index is the average of ‘*r*’ values of all the parameters evaluated in the research study. The acceptable limits correlate to a score of 5.

IPP= The average of the “*r*” values of all the *n*° values (> 5). **Eq 9**

$$Reliability factor \left( f \right)=\frac{Polygon area}{Circle area} \mathbf{Eq}\mathbf{10}$$

The value of *f* = 0.952 for 12 parameters.

**Index of Good Compressibility and Bucco-dispersibility (IGCB):**

The index of good compressibility (ICG) is the product of the parameter profile index and reliability factor.

$$Good Compressibility Index=ICG=IPP*f \left( >5 \right). \mathbf{Eq}\mathbf{11}$$

The flurbiprofen - ODT powder blends were calculated by six different incidences having fifteen (15) parameters. Index Parameter (IP) and Profile Index Parameter (IPP) were described earlier in the study while the Index of Good Compressibility and Bucco-dispersibility (IGCB) is the product of the parameter profile index and reliability factor was measured as follows:

$$Good Compressibility and Buccodispersibility Index=IGCB=IPP*f \mathbf{Eq 12}$$

The value of *f* = 0.971 for 15 parameters. After measuring all the parametric indices, the results were calculated by a radar chart, and the final ICGB value for all powder blends (F1-F9). The minimum percentages of ludipress required for the rectification of the maximum deficient incidence parameter value of flurbiprofen were estimated using the below equation:

 **Eq 14**

Where,

‘CP’ = Percentage (%) of corrective excipient required to correct the particular index in API.

‘RE’ = is the parametric incidence value of the corrective excipient.

‘RP’ = is the parametric incidence value of the API essential to be corrected.

‘R’ = is the desired incidence parameter value.

**Table S1**

**Basic Parameters Determination Following SeDeM-ODT Tool**

| ***INCIDENCE*** | ***PARAMETERS*** | ***LIMIT VALUE (v)*** | ***FACTORS APPLIED TO v*** | ***RADIUS***  ***(r)*** |
| --- | --- | --- | --- | --- |
| ***DIMENSION*** | ***Bulk Density (Da)*** | 0-1 g /ml | 10 *v* | 0-10 |
|  | ***Tapped Density (Dc)*** | 0-1 g/ml | 10 *v* | 0-10 |
| ***COMPRESSIBILITY*** | ***Inter-Particle Prorosity (Ie)*** | 0-1.2 | 10 *v* / 1.2 | 0-10 |
|  | ***Carr Index (IC)*** | 0-50 % | *v* / 5 | 0-10 |
|  | ***Cohesion Index (Icd)*** | 0-200 % | *v* / 20 | 0-10 |
| ***FLOWABILITY/ POWDER FLOW*** | ***Hausner Ratio (IH)*** | 1-3 | 5 (3- *v)* | 0-10 |
|  | ***Angle Of Repose (θ)*** | 50-0 (º) | 10- (*v / 5)* | 0-10 |
|  | ***Powder Flow (t”)*** | 20-0 (s) | 10- (*v / 2)* | 0-10 |
| ***LUBRICITY / STABILITY*** | ***Loss on Drying (% HR)*** | 0-10 (%) | 10- *v* | 0-10 |
|  | ***Hygroscopicity (%H)*** | 20-0 (%) | 10- (*v / 2)* | 0-10 |
| ***LUBRICITY / DOSAGE*** | ***Particle Size (%Pf)*** | 50-0 (%) | 10- (*v / 5)* | 0-10 |
|  | ***Homogeneity Index (Iθ)*** | 0-2x10^-2^ | 500 *v* | 0-10 |
| ***DISGREGABILITY*** | ***Effervescence*** | 0-5 (min) | (5- *v*) *2 | 0-10 |
|  | ***Disintegration time with disk (DCD)*** | 0-3 (min) | (3- *v*) *3.333 | 0-10 |
|  | ***Disintegration time without disk (DSD)*** | 0-3 (min) | (3- *v*) *3.333 | 0-10 |

**Table S2**

**Selection of variables, Levels and Responses for Central Composite Design (CCD)**

| **Independent Variables** | **Levels** |  |
| --- | --- | --- |
|  | **High** | **Low** |
| X_1_ = Ludipress (%) | 55 | 49 |
| X_2_ = Croscarmellose Sodium (%) | 5 | 1 |
| **Dependent Variables** | **Constraints** | |
| **R_1_ = Hardness** | **3kg – 5 kg** | |
| **R_2_ = Friability** | **< 1 %** | |
| **R_3_ = Disintegration Test** | **< 3 min** | |

Table S3

Probability Value and Coded Equations of Selected Responses

| Responses | *p* value |
| --- | --- |
| R_1_ (Disintegration) | 0.0003 |
| R_2_ (Hardness) | 0.0167 |
| R_3_ (Friability) | 0.0442 |
| Coded Equations for Responses | |
| R_1_ =$\boldsymbol{+12.11+0.4786}\boldsymbol{A-2.79*B}$ | |
| R_2_ =+$\mathbf{4.34+0.6399*A+0.0207*B+0.0825*AB+0.2700*A}^{\begin{aligned} \boldsymbol{2} \\ \end{aligned}}\boldsymbol{+0.4775*}\boldsymbol{B}^{\boldsymbol{2}}$ | |
| R_3_ = $\boldsymbol{+0.5633-0.0226*A-0.0239*B}$ | |

**TABLE S4**

**STATISTICAL MODEL SUMMARY FOR DISINETRGARTION TEST**

| **Source** | **Sum of Squares** | **df** | **Mean Square** | **F-value** | **p-value** |  |
| --- | --- | --- | --- | --- | --- | --- |
| **Model** | 64.07 | 2 | 32.03 | 39.89 | 0.0003 | significant |
| A-LUDIPRESS | 1.83 | 1 | 1.83 | 2.28 | 0.1817 |  |
| B-CROSCARMELLOSE SODIUM | 62.24 | 1 | 62.24 | 77.49 | 0.0001 |  |
| **Residual** | 4.82 | 6 | 0.8032 |  |  |  |
| **Cor Total** | 68.89 | 8 |  |  |  |  |

**TABLE S5**

**STATISTICAL MODEL SUMMARY FOR HARDNESS TEST**

| **Source** | **Sum of Squares** | **df** | **Mean Square** | **F-value** | **p-value** |  |
| --- | --- | --- | --- | --- | --- | --- |
| **Model** | 3.98 | 5 | 0.7950 | 19.82 | 0.0167 | significant |
| A-LUDIPRESS | 3.28 | 1 | 3.28 | 81.66 | 0.0029 |  |
| B-CROSCARMELLOSE SODIUM | 0.0034 | 1 | 0.0034 | 0.0854 | 0.7891 |  |
| AB | 0.0272 | 1 | 0.0272 | 0.6787 | 0.4704 |  |
| A² | 0.2121 | 1 | 0.2121 | 5.29 | 0.1050 |  |
| B² | 0.6633 | 1 | 0.6633 | 16.54 | 0.0268 |  |
| **Residual** | 0.1203 | 3 | 0.0401 |  |  |  |
| **Cor Total** | 4.10 | 8 |  |  |  |  |

**TABLE S6**

**STATISTICAL MODEL SUMMARY FOR FRIABILITY TEST**

| **Source** | **Sum of Squares** | **df** | **Mean Square** | **F-value** | **p-value** |  |
| --- | --- | --- | --- | --- | --- | --- |
| **Model** | 0.0087 | 2 | 0.0043 | 5.49 | 0.0442 | significant |
| A-LUDIPRESS | 0.0041 | 1 | 0.0041 | 5.17 | 0.0633 |  |
| B-CROSCARMELLOSE SODIUM | 0.0046 | 1 | 0.0046 | 5.80 | 0.0527 |  |
| **Residual** | 0.0047 | 6 | 0.0008 |  |  |  |
| **Cor Total** | 0.0134 | 8 |  |  |  |  |

**TABLE S7**

**FLURBIPROFEN – ODT TABLETS ACCELERATED STABILITY TESTING REPORT USING MINITAB SOFTWARE**

**Factor Information**

| **Factor** | **Type** | **Number of Levels** | **Levels** |
| --- | --- | --- | --- |
| Batch | Fixed | 9 | FLURB-F1, FLURB-F2, FLURB-F3, FLURB-F4, FLURB-F5, FLURB-F6, FLURB-F7, FLURB-F8, FLURB-F9 |

**Model Selection with α = 0.25**

| **Source** | **DF** | **Seq SS** | **Seq MS** | **F-Value** | **P-Value** |
| --- | --- | --- | --- | --- | --- |
| Month | 1 | 6.820 | 6.8204 | 25.94 | 0.001 |
| Batch | 8 | 6.719 | 0.8399 | 3.19 | 0.052 |
| Month*Batch | 8 | 2.718 | 0.3397 | 1.29 | 0.353 |
| Error | 9 | 2.366 | 0.2629 |  |  |
| Total | 26 | 18.624 |  |  |  |
| **Source** | **DF** | **Seq SS** | **Seq MS** | **F-Value** | **P-Value** |
| Month | 1 | 6.820 | 6.8204 | 22.81 | 0.000 |
| Batch | 8 | 6.719 | 0.8399 | 2.81 | 0.035 |
| Error | 17 | 5.084 | 0.2991 |  |  |
| Total | 26 | 18.624 |  |  |  |

*Terms in selected model: Month, Batch*

**Model Summary**

| **S** | **R-sq** | **R-sq(adj)** | **R-sq(pred)** |
| --- | --- | --- | --- |
| 0.546869 | 72.70% | 58.25% | 30.52% |

**Coefficients**

| **Term** | **Coef** | **SE Coef** | **T-Value** | **P-Value** | **VIF** |
| --- | --- | --- | --- | --- | --- |
| Constant | 105.753 | 0.166 | 635.51 | 0.000 |  |
| Month | -0.2052 | 0.0430 | -4.78 | 0.000 | 1.00 |
| Batch |  |  |  |  |  |
| FLURB-F1 | -0.614 | 0.298 | -2.06 | 0.055 | 1.78 |
| FLURB-F2 | -0.211 | 0.298 | -0.71 | 0.489 | 1.78 |
| FLURB-F3 | -0.124 | 0.298 | -0.42 | 0.682 | 1.78 |
| FLURB-F4 | -0.844 | 0.298 | -2.84 | 0.011 | 1.78 |
| FLURB-F5 | 0.579 | 0.298 | 1.95 | 0.068 | 1.78 |
| FLURB-F6 | -0.201 | 0.298 | -0.67 | 0.509 | 1.78 |
| FLURB-F7 | 0.429 | 0.298 | 1.44 | 0.167 | 1.78 |
| FLURB-F8 | 0.643 | 0.298 | 2.16 | 0.045 | 1.78 |
| FLURB-F9 | 0.343 | 0.298 | 1.15 | 0.266 | * |

**TABLE S8**

**SHELF LIFE ESTIMATION**

Lower spec limit = 90   Upper spec limit = 110
Shelf life = time period in which you can be 95% confident that at least 50% of response is within spec limits.

| **Batch** | **Shelf Life** |
| --- | --- |
| FLURB-F1 | 51.924 |
| FLURB-F2 | 53.292 |
| FLURB-F3 | 53.586 |
| FLURB-F4 | 51.144 |
| FLURB-F5 | 55.971 |
| FLURB-F6 | 53.326 |
| FLURB-F7 | 55.462 |
| FLURB-F8 | 56.186 |
| FLURB-F9 | 55.169 |
| Overall | 51.144 |


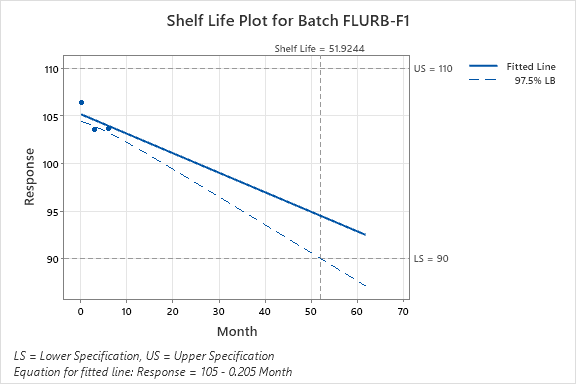


**FIG S1: SHELF LIFE PLOT FOR FORMULATION 1**


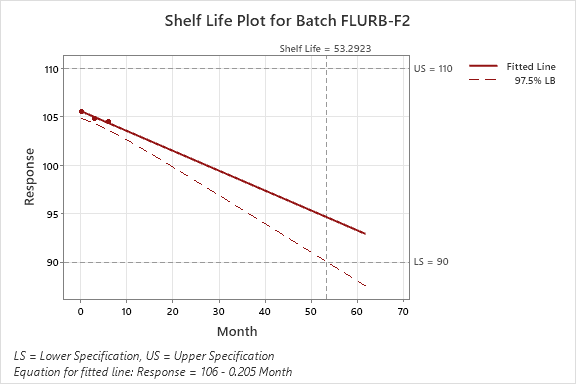


**FIG S2: SHELF LIFE PLOT FOR FORMULATION 2**


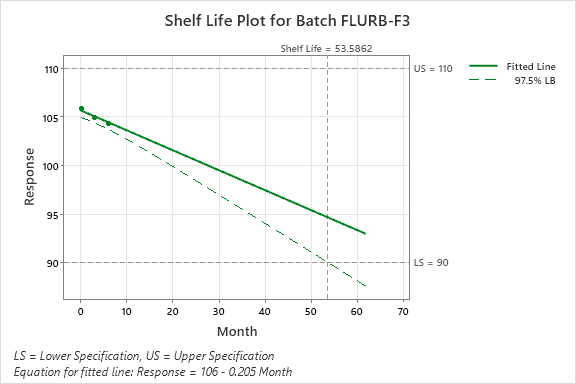


**FIG S3: SHELF LIFE PLOT FOR FORMULATION 3**


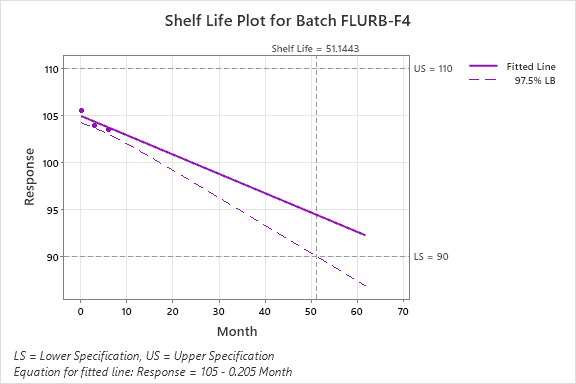


**FIG S4: SHELF LIFE PLOT FOR FORMULATION 4**


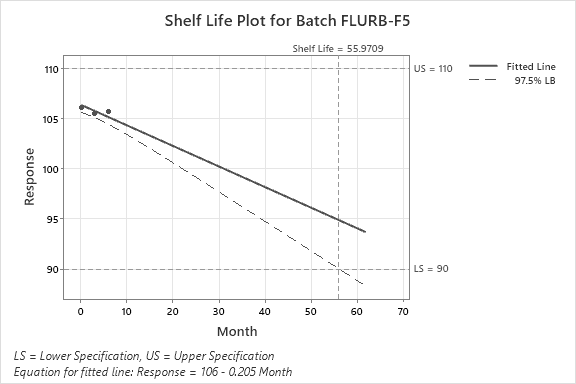


**FIG S5: SHELF LIFE PLOT FOR FORMULATION 5**


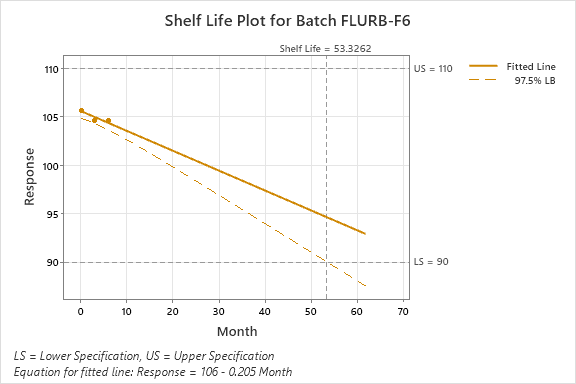


**FIG S6: SHELF LIFE PLOT FOR FORMULATION 6**


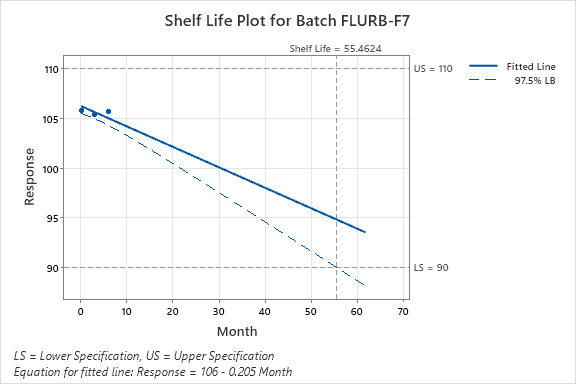


**FIG S7: SHELF LIFE PLOT FOR FORMULATION 7**


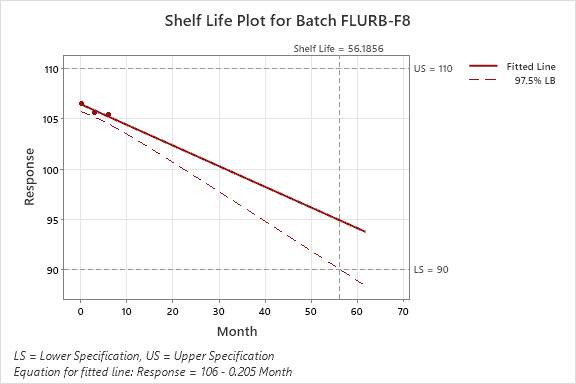


**FIG S8: SHELF LIFE PLOT FOR FORMULATION 8**


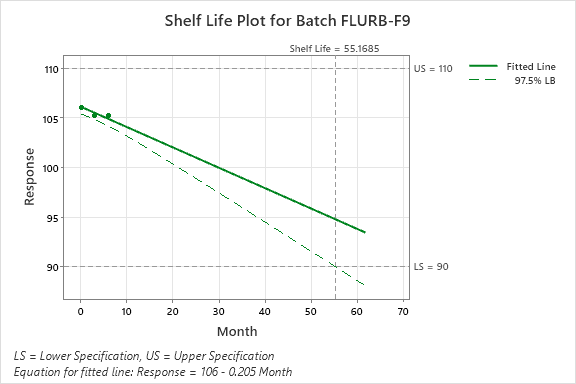


**FIG S9: SHELF LIFE PLOT FOR FORMULATION 9**
